# Quantitative analysis of the Stratus optical coherence tomography fast macular thickness map reports

**DOI:** 10.4103/0301-4738.60085

**Published:** 2010

**Authors:** Amitha Domalpally, Ronald P Danis, Dawn Myers, Christina N Kruse

**Affiliations:** Department of Ophthalmology and Visual Sciences, Fundus Photograph Reading Center, University of Wisconsin, Madison

**Keywords:** Centerpoint thickness, optical coherence tomography artifacts, Stratus optical coherence tomography

## Abstract

The cross sectional optical coherence tomography images have an important role in evaluating retinal diseases. The reports generated by the Stratus fast macular thickness scan protocol are useful for both clinical and research purposes. The centerpoint thickness is an important outcome measure for many therapeutic trials related to macular disease. The data is susceptible to artifacts such as decentration and boundary line errors and could be potentially erroneous. An understanding of how the data is generated is essential before utilizing the data. This article describes the interpretation of the fast macular thickness map report, assessment of the quality of an optical coherence tomography image and identification of the artifacts that could influence the numeric data.

The introduction of Stratus optical coherence tomogram (OCT) (Zeiss, Meditec, Dublin, CA) has advanced the knowledge and understanding of retinal diseases.[1] Apart from being used for detailed morphological evaluation,[2,3] OCT is also used for quantitative analysis of retinal thickness. The numeric plots are used for quantification of macular disease[4,5] and monitoring progression.[6,7] OCT-measured retinal thickness value is an important outcome measure in many therapeutic intervention trials.[8–10]

The Stratus OCT comes with various scanning programs; the most commonly used protocols for retinal pathologies are line scan, cross hair scan, the macular thickness map protocol (MTM) and the fast macular thickness map protocol (FMTM).[11] Both MTM and FMTM consist of six radial line scans which cover a diameter of 6 mm and generate thickness reports which are extremely useful for quantitative analysis. The FMTM scans have a low resolution (128 A-scans/B-scan) and are less time-consuming (1.92 sec for the entire scan) whereas the MTM scans provide high-resolution images (512 A-scans/B-scan) with each radial line taking 1.28 sec. Due to its time efficiency, the FMTM protocol is frequently preferred.

Longitudinal assessment of the foveal thickness on OCT is an important outcome measure for many therapeutic trials. The fundus photograph reading center (FPRC), Madison WI, USA, serves as a central laboratory to receive and analyze OCTs for multicenter clinical trials. The scans are taken by trained and certified operators and evaluated at the reading center by ocular disease evaluators (ODE). Quality of the OCT is one of the most important evaluation procedures used by the ODE since it determines if the software-generated numeric data is erroneous or not. Table 1 summarizes the artifacts frequently seen in OCTs. Artifacts generated by the software are common and could potentially produce erroneous data.[12–14] Manual remeasurement of foveal thickness is performed in scans with faulty centerpoint thickness (CPT). Over 30,000 OCT scans are evaluated annually at the reading center and nearly 30% need manual remeasurement.

**Table 1:** Artifacts in the fast macular thickness map protocol of the stratus optical coherence tomogram

	Artifact	Interpretation
Operator-dependent	Decentration	Misidentification of foveal center
	Z offset error	Faulty scan registration; the B-scan is vertically displaced in the OCT window
	Cut edge artifacts	Edge of the scan truncated
Operator independent	Boundary line errors	Inner and/or Outer boundary lines do not correspond to ILM or RPE, respectively
	Alignment artifacts[17]	Distortion/flattening of morphology in AMD/central serous chorioretinopathy following automated retinal thickness (single eye) analysis on Stratus software
	Artifacts due to low signal strength	Layers on the B-scan not clearly seen

An understanding of the software algorithms and how each of the elements of the report is generated is essential for interpreting the thickness measurements. The purpose of this article is to assist with interpretation of the reports generated by the FMTM protocol and understanding of the artifacts generated by the OCT.

## The fast macular thickness map protocol

**Figure 1:**
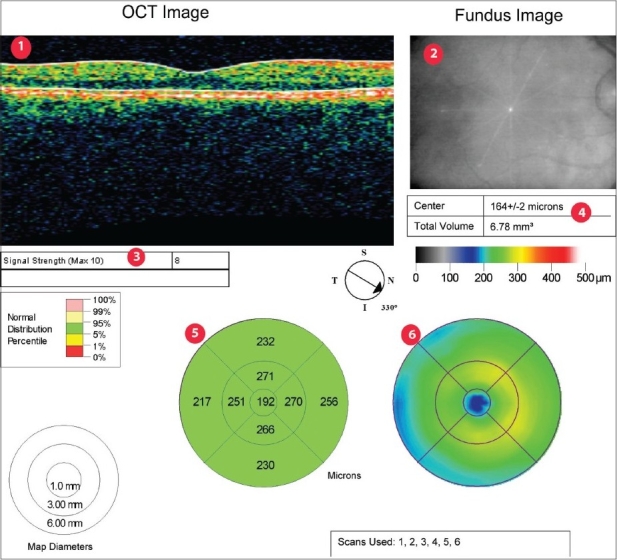
Retinal map analysis report showing (1) Optical coherence tomogram image, B-Scan (2) Fundus Image with radial lines showing scan location (3) Signal strength (4) Centerpoint thickness (5) Numeric plot (6) Pseudocolor map

**Figure 2:**
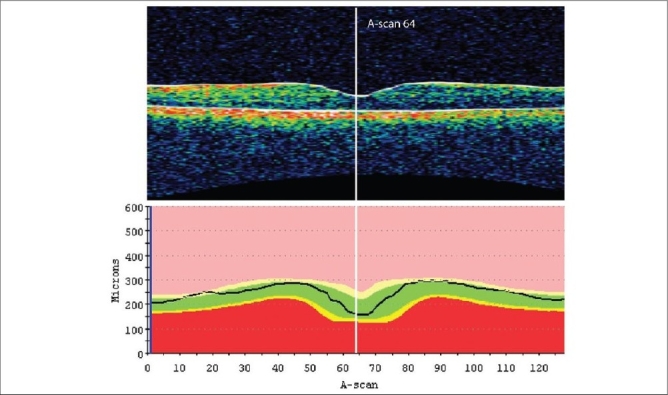
Retinal thickness reports showing the center of the macula corresponding to the centerpoint of the scan or A-scan 64. Centerpoint thickness is measured at A-scan 64

**Figure 3:**
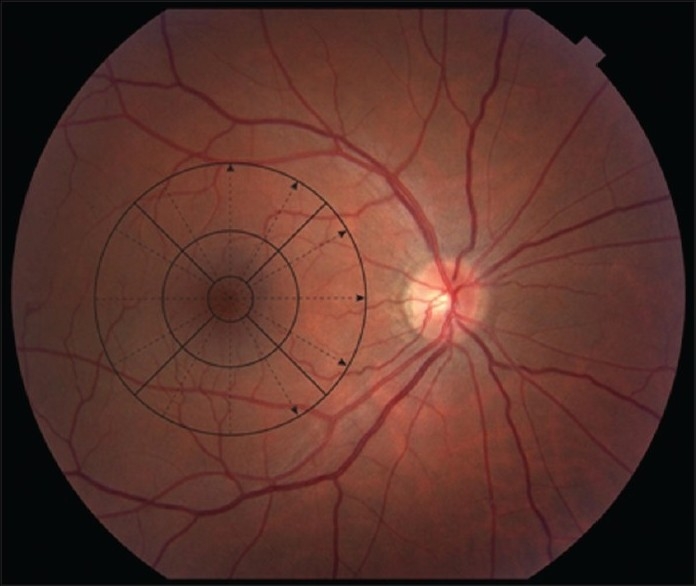
Right eye fundus image showing location of optical coherence tomogram-modified early treatment diabetic retinopathy study grid. The dotted arrows represent the direction of the six radial scan lines and their relation to the subfields of the grid

The combined data from the six radial scans of the FMTM scanning protocol is represented in the FMTM retinal map analysis report. The individual component B-scans are available on retinal thickness reports. The map report should always be analyzed in conjunction with the six individual thickness reports. The map report has six components to it [Fig. 1]. (1) The B-scan (OCT image) is a truncated image of the scan with the least signal intensity. (2) The fundus image is taken after the scan is complete and can show the location of the radial scan lines and the centerpoint (intersection of the six lines). (3) The signal strength is a quality measurement which employs the signal/noise ratio on a scale of 1 to 10. Signal strength tends to correlate with the contrast between layers of the retina and thus the boundary line algorithm of the machine. A signal strength less than 5 frequently indicates poor scan quality. The confidence message given by the software (available in the newer software versions) informs the clinician of the probability of artifacts affecting the data. (4) The CPT is given in microns with a standard deviation and the total volume is given in mm^3^. The six radial B-scans are comprised of 128 A-scans each and the centerpoint of each of these scans is represented by A-scan no. 64 [Fig. 2]. The algorithm identifies A-scan 64 and measures the retinal thickness (distance between the two boundary lines) at this point. The mean and standard deviation is derived from the thickness measurements of the six scans and is presented as CPT with a standard deviation. (5) A numeric plot provides nine mean thickness values (one for each of the nine subfields) and is similar to the early treatment diabetic retinopathy study (ETDRS) grid. (6) A pseudocolor topographic map is a representation of the retinal thickness using a color scale. The radial lines of the scan are represented in the subfields as shown in Fig. 3.

The quality of the OCT scan needs to be evaluated before interpreting any of the components. Fig. 1 demonstrates an example of a good quality OCT image. The B-scan has correctly placed boundary lines, the radial scan lines on the fundus image seem to intersect at the macula, the signal strength is good (>5). The standard deviation of the CPT is less than 10% of the CPT. The fovea (blue area) is positioned in the central subfield of the pseudocolor map. The numeric data available on the report of a good quality OCT can be readily accepted. Poor quality OCT scans have artifacts that affect their numeric data. The CPT can be remeasured using commercially available software (Stratus review software); however, the subfield values cannot be reclaimed. Table 1 gives a list of possible artifacts with the Stratus OCTs. Two major errors that could affect the numeric data and occur quite commonly are boundary line errors and decentration.

## Boundary line errors

**Figure 4:**
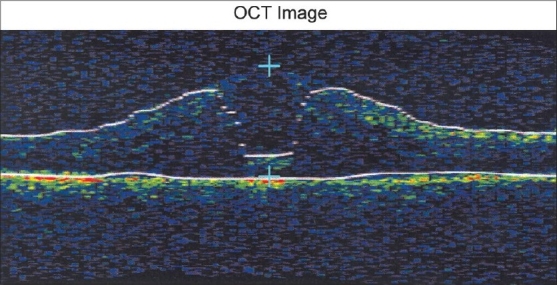
Boundary line errors of the center of the macula. The centerpoint thickness is remeasured using software calipers (blue cursors)

There are two boundary lines identified by the software in a cross-sectional retinal scan-the first hyper-reflective band is the internal limiting membrane (ILM) and the second hyper-reflective band is considered to be the retinal pigment epithelium (RPE). Studies have shown that the second narrow hyper-reflective band in normal eyes is the photoreceptor inner segment-outer segment (IS-OS) junction and that the true RPE is missed by the segmentation algorithm.[15,16] Errors in the boundary line could occur due to misidentification of either the ILM or RPE. Vitreous detachment, macular holes, epiretinal membranes, and large cysts are some of the morphological abnormalities that could create boundary line errors of the ILM. The RPE boundary could be erroneously drawn in eyes with neovascular lesions, or other abnormalities which would create hyper-reflectivity such as blood and hard exudates. Errors of both layers could occur in images with low signal strength or poor scan registration (Z offset error). Retinal thickness is calculated as the distance between the two boundary lines. Errors in the boundary lines could therefore adversely affect the measurements. If the error involves the centerpoint of the scan, the CPT is affected. In such cases, the Stratus review software can be used to remeasure CPT by placing the software calipers at the true boundary lines of the centerpoint [Fig. 4]. If the boundary line error affects any other part of the scan, then the subfield values of the corresponding radial scan are incorrect.

**Figure 5:**
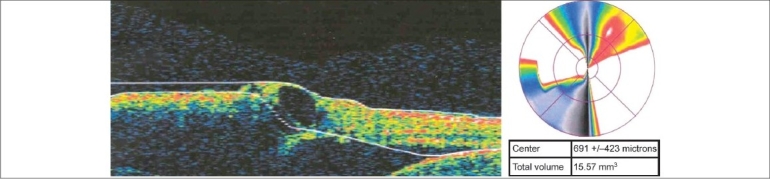
Wedge artifact seen on pseudocolor map and the high standard deviation are clues for presence of boundary line errors. The underlying B-scan shows large boundary line errors

Boundary line errors can easily be identified using the six individual B-scans. Wedge or bowtie artifacts on the pseudocolor map and a high standard deviation of the CPT are additional clues to the presence of this artifact [Fig. 5]. On occasion, rescanning may avoid this artifact but mostly, these errors are unavoidable and result in lost data. The software versions have been upgraded to correct boundary lines and automatically recalculate the subfield values.

## Decentration

CPT is an important parameter for evaluation in macular disease because it represents foveal thickness. An important aspect to quality assessment is to confirm whether the CPT truly represents the foveal thickness. If the radial lines of the scan pattern have not been centered on the fovea, the scan is considered decentered and the CPT would not represent the foveal thickness. The center of the macula corresponds to A-scan 64 as shown in Fig. 2 and the central subfield of the numeric plot is assumed to correspond to the macula in well-centered scans as shown in Fig. 3. In decentered scans, the CPT does not represent true foveal thickness. The numeric plot is also shifted and the values of the subfields do not represent their presumed location on the fundus.

**Figure 6:**
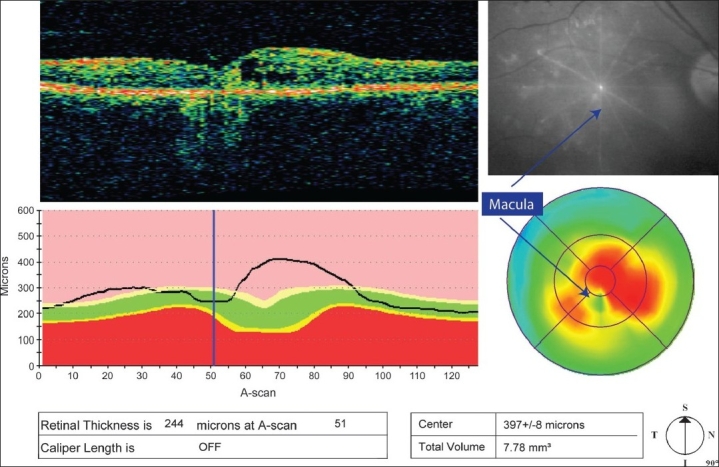
Decentration artifact. The location of the fovea on the B-scan is shifted from A-scan 64 (blue line). The fundus image shows that the intersecting point of the radial lines does not correspond to the anatomical location of the center of the macula (blue arrows). The area representing the center of the macula is not located within the central subfield of the pseudocolor map. The standard deviation is not high. The optical coherence tomogram-measured centerpoint thickness is 397μ but the true foveal thickness remeasured is 244μ

Decentration artifact can be assessed using a combination of clues [Fig. 6]. In scans with a visible foveal depression, if the fovea is not located at A-scan 64 the scan is assumed to be decentered. The OCT image on the map report is truncated and should not be used for assessing decentration. The six underlying B-scans from the retinal thickness reports can be used to evaluate decentration. The radial lines should intersect at the center of the macula in the fundus image. However, it should be kept in mind that the fundus image is taken after the OCT scan is completed. In thin retinas with normal foveal depression, the fovea is represented by a blue circular area on the pseudocolor map and is located within the central subfield if well centered. The standard deviation may or may not be high in decentered scans. If the foveal depression is not clearly visible, alternative clues are used to identify the fovea such as change in the reflectivity of the inner layer due to loss of ganglion cell layer at the fovea, proximity of the largest cyst in cystoid macular edema (assumed to be close to the fovea), and examination of the cross hair scans which may better display the fovea and can be used to reference the location on the fast macular scans.

Decentration is mostly an operator-dependent error. Poor patient fixation or inability to identify the fovea in a distorted retina could be contributory factors. However, if identified, rescanning could help avoid this artifact. In decentered scans, remeasurement of foveal thickness using the software calipers (Stratus review software) is a possibility. The retinal thickness report with an identifiable fovea is selected and the calipers are placed at the inner and outer boundary lines of the point identified as fovea. This gives the true foveal measurement. The remaining data in the subfields cannot be reclaimed.

In summary, artifacts on OCTs performed using the FMTM scan protocol of the Stratus OCT are common. Identification of operator-dependent artifacts and retaking images if required reduces the frequency of poor quality OCTs in clinical trials. Simple measures such as centering the scan in a well-dilated pupil, focus and z offset adjustment, and having the patient close their eyes to wet the cornea before taking the image also improves image quality. Poor quality OCTs with erroneous numeric data may still be useful for qualitative analysis of morphological abnormalities.
